# Multi-Round versus Real-Time Delphi survey approach for achieving consensus in the COHESION core outcome set: a randomised trial

**DOI:** 10.1186/s13063-023-07388-9

**Published:** 2023-07-19

**Authors:** Fiona A. Quirke, Malcolm R. Battin, Caitlin Bernard, Linda Biesty, Frank H. Bloomfield, Mandy Daly, Elaine Finucane, David M. Haas, Patricia Healy, Tim Hurley, Sarah Koskei, Shireen Meher, Eleanor J. Molloy, Maira Niaz, Elaine Ní Bhraonáin, Christabell Omukagah Okaronon, Farhana Tabassum, Karen Walker, James R. H. Webbe, Matthew J. Parkes, Jamie J. Kirkham, Declan Devane

**Affiliations:** 1 Health Research Board Neonatal Encephalopathy PhD Training Network (NEPTuNE), Dublin, Ireland; 2 Health Research Board – Trials Methodology Research Network (HRB-TMRN), Galway, Ireland; 3 School of Nursing and Midwifery, University of Galway, Galway, Ireland; 4 Department of Newborn Services, Auckland District Health Board, Auckland, New Zealand; 5 Department of Obstetrics and Gynecology, Indiana University, Indianapolis, IN USA; 6 Evidence Synthesis Ireland, University of Galway, Galway, Ireland; 7 Qualitative Research in Trials Centre (QUESTS), University of Galway, Galway, Ireland; 8 Liggins Institute, University of Auckland, Private Bag 92019, Auckland, 1142 New Zealand; 9 Advocacy and Policymaking, Irish Neonatal Health Alliance, Wicklow, Ireland; 10 Department of Paediatric and Child Health, Trinity College Dublin, Tallaght University Hospital (TUH), Dublin, Ireland; 11 Moi University, Eldoret, Kenya; 12 Birmingham Women’s and Children’s NHS Foundation Trust, Birmingham, UK; 13 Paediatrics and Child Health, Trinity College Dublin, Dublin, Ireland; 14 Department of Neonatology, Children’s Hospital Ireland at Crumlin and Tallaght, Coombe Women and Infants University Hospital, Dublin, Ireland; 15 Department of Paediatrics & Child Health, Aga Khan University, Karachi, Pakistan; 16 Family Support Liaison, Irish Neonatal Health Alliance, Wicklow, Ireland; 17 AMPATH, Eldoret, Kenya; 18 Centre of Excellence in Women and Child Health, Aga Khan University, Karachi, Pakistan; 19 Department of Newborn Care, Royal Prince Alfred Hospital, Sydney, Australia; 20 University of Sydney, Sydney, Australia; 21 The George Institute for Global Health, Sydney, Australia; 22 Council of International Neonatal Nurses, Sydney, Australia; 23 Academic Neonatal Medicine, Imperial College London, London, UK; 24 Centre for Statistics in Medicine, Nuffield Department of Orthopaedics Rheumatology and Musculoskeletal Science, University of Oxford, Oxfordshire, UK; 25 Centre for Biostatistics, The University of Manchester, Manchester Academic Health Science Centre, Manchester, UK; 26 Cochrane Ireland, University of Galway, Galway, Ireland

**Keywords:** Real-Time Delphi, Multi-round Delphi, Core outcome set, Randomised trial, Delphi survey, Consensus

## Abstract

**Background:**

Delphi surveys are commonly used to prioritise critical outcomes in core outcome set (COS) development. This trial aims to compare a three-round (Multi-Round) Delphi (MRD) with a Real-Time Delphi (RTD) in the prioritisation of outcomes for inclusion in a COS for neonatal encephalopathy treatments and explore whether ‘feedback’, ‘iteration’, and ‘initial condition’ effects may occur in the two survey methods.

**Methods:**

We recruited 269 participants (parents/caregivers, healthcare providers and researchers/academics) of which 222 were randomised to either the MRD or the RTD. We investigated the outcomes prioritised in each survey and the ‘feedback’, ‘iteration’, and ‘initial condition’ effects to identify differences between the two survey methods.

**Results:**

In the RTD, *n* = 92 participants (83%) fully completed the survey. In the MRD, *n* = 60 participants (54%) completed all three rounds. Of the 92 outcomes presented, 26 (28%) were prioritised differently between the RTD and MRD. Significantly fewer participants amended their scores when shown stakeholder responses in the RTD compared to the MRD (‘feedback effect’). The ‘iteration effect’ analysis found most experts appeared satisfied with their initial ratings in the RTD and did not amend their scores following stakeholder response feedback. Where they did amend their scores, ratings were amended substantially, suggesting greater convergence. Variance in scores reduced with subsequent rounds of the MRD (‘iteration effect’). Whilst most participants did not change their initial scores in the RTD, of those that did, later recruits tended to align their final score more closely to the group mean final score than earlier recruits (an ‘initial condition’ effect).

**Conclusion:**

The feedback effect differed between the two Delphi methods but the magnitude of this difference was small and likely due to the large number of observations rather than because of a meaningfully large difference. It did not appear to be advantageous to require participants to engage in three rounds of a survey due to the low change in scores. Larger drop-out through successive rounds in the MRD, together with a lesser convergence of scores and longer time to completion, indicate considerable benefits of the RTD approach.

**Trial registration:**

NCT04471103. Registered on 14 July 2020.

**Supplementary Information:**

The online version contains supplementary material available at 10.1186/s13063-023-07388-9.

## Background

A core outcome set (COS) is an agreed, standardised collection of outcomes that key stakeholders have agreed should be measured as a minimum in all trials and other studies for specific health conditions [1]. During COS development, stakeholder input is achieved through several methods, including interviews, focus groups, Delphi surveys and consensus meetings [1]. Delphi surveys are the most common method of achieving consensus, either alone or in combination with other techniques [2].

The Delphi method aims to incorporate the positive aspects of group participation, such as input from different viewpoints and expertise, without the negative constraints of social interactions in face-to-face settings [3, 4].

The Delphi approach is characterised by several factors, including ‘anonymity, iteration, controlled feedback, and statistical aggregation of group response’ [4].

A Delphi survey also allows participants to contribute views or new ideas for survey questions. This information can be moderated by the survey administrator and presented to other participants for comment or voting [4].

In the context of COS development, the Delphi survey involves iterative rounds of an online survey whereby outcomes are listed and participants are asked to rate their importance, typically on a 9-point Likert scale [1]. Many previous COSs have included at least two rounds of a Delphi survey, with many others including three rounds [1]. The ratings given by each stakeholder group for an outcome in the previous round are presented in subsequent rounds. This feedback allows participants to consider other stakeholders’ opinions before they are given the option to re-rate the outcome or keep their rating the same.

Although the Delphi method is commonly used to achieve consensus on what outcomes are ‘core’ in COS development, shortfalls with the approach have been noted [5]. Among these are the length of the overall process when engaging participants in two or more rounds of a Delphi survey and delays in presenting the participants with feedback, both of which can contribute to loss of interest and reduced satisfaction among participants [5].

In recent years, the concept of a ‘Real-Time’ Delphi (RTD) has emerged. The functionality of a RTD can potentially improve the shortfalls of engaging in a long Delphi survey process with multiple rounds [6, 7]. Some groups developing RTD functionality describe the group response feedback as being available when the participant revisits the survey [7]. In contrast, others describe feedback, in the form of group responses, as being provided immediately after a participant has answered a question [8].

Gnatzy et al. [6] have compared a RTD approach with a Multi-Round Delphi (MRD) survey. Although different datasets were used and surveys were carried out at different times, with different participants, and in different locations, their results indicated no significant differences between the two survey methods (i.e. no significant differences in feedback effect or iteration effect) and the final results (i.e. there were no differences in how the sample of questions was answered by the two surveys) were not impacted by differences in the survey methods. However, given that the study conducted by Gnatzy et al. [6] compared surveys with different datasets, further research is needed to compare RTD with MRD survey approaches in a randomised trial using the same dataset.

## Methods

### Aim

The aim of this study was to identify if different outcomes are prioritised when using a Multi-Round compared to a RTD survey approach in developing a COS for treatments of neonatal encephalopathy and to compare the ‘feedback’, ‘iteration’, and ‘initial condition’ effects (defined by Gnatzy et al. [6]) in the two survey methods to examine if differences exist between the two approaches.

### Design

This study involves a two-arm, parallel randomised trial. In reporting the protocol for this study, we followed the SPIRIT 2013 checklist [9, 10].

The outcome of the COS study [11] has been published elsewhere.

The reporting of this trial follows the CONSORT Checklist [12] (Fig. 3) (Additional file 1: Appendix 1).

### Participants

We recruited stakeholders with expertise in neonatal encephalopathy to develop a COS for treating neonatal encephalopathy [9, 13]. Stakeholders included parents of infants diagnosed with and treated for neonatal encephalopathy or caregivers who care for the infant, healthcare providers and researchers or academics with expertise in neonatal encephalopathy research.

### Interventions

**Fig. 1:**
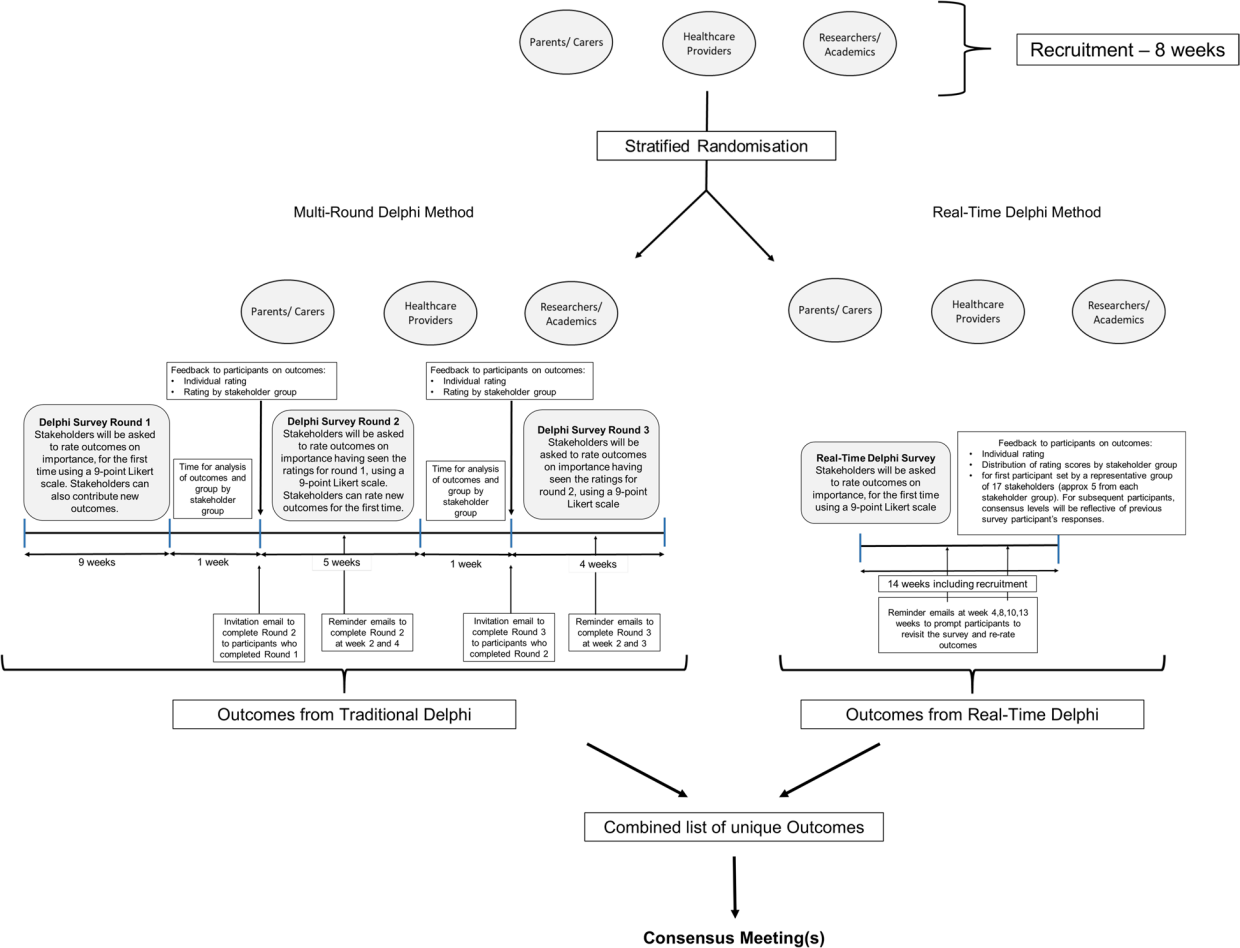
Timeline of randomised trial

Respondents were randomised to answer the online Delphi survey in either a three-round e-Delphi format or a Real-Time e-Delphi format (Fig. 1). The same list of outcomes was presented to participants of both surveys. Participants were not blinded to the intervention arm as instruction videos were provided to participants to describe the steps of the survey and how to complete the survey. The lead researcher was also not blinded to the intervention arm as they were required to access the system database to retrieve data for analyses, generate reports, track participation and other administrative tasks.

#### Real-Time Delphi method

To ensure the RTD was populated with feedback responses representative of the stakeholder ratings when it went live, we initially recruited 34 participants. These participants were randomised to each survey arm (*n* = 17 in each survey) in August 2021 and were representative of each stakeholder group (parents/caregivers, five healthcare providers, and five researchers/academics). These participants were also included in the final survey numbers. Once the survey was live to other participants, the first 17 participants recruited to the RTD survey were emailed and invited to revisit the survey to see stakeholder feedback graphs and amend their rating for each outcome if they wished. Recruitment and duration of the surveys are reported in Fig. 1. Overall, the MRD was live, including recruitment, for 20 weeks compared to 14 weeks for the RTD survey.

In our RTD survey, participants initially rate the outcome without seeing other stakeholders’ responses. Once they have rated an outcome, the survey page refreshes and the response graphs of all stakeholder groups are displayed for the participant. At this point, the participant can change their rating or keep it the same before moving on to rate the next outcome. As in Gordon and Peases’ model [7], participants can revisit and re-rate outcomes as often as they wish during the survey period.

The RTD survey presented 87 outcomes for rating. These outcomes were identified from a systematic review of randomised trials of interventions for the treatment of neonatal encephalopathy [14] and qualitative interviews with parents/caregivers from high-income countries (HiCs) and low- to middle-income countries (LMiCs) [15]. Five additional outcomes were added to the survey based on stakeholder feedback. The surveys also sought basic demographic information from participants to ensure contributions from stakeholders in different countries were obtained.

**Fig. 2:**
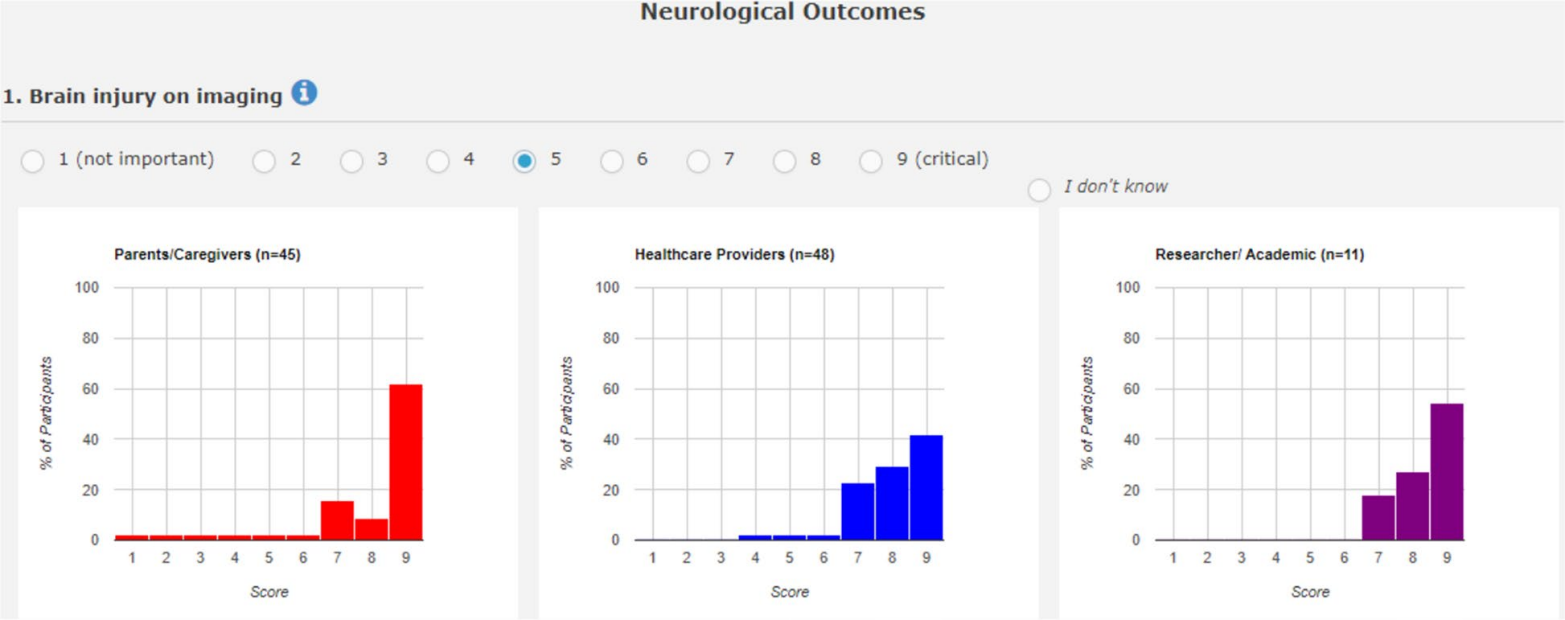
Example of stakeholder rating responses in bar chart format

Participants were asked to rate the importance of each outcome for inclusion in the COS for neonatal encephalopathy treatments on a 9-point Likert scale, typically used in COS development [1], in terms of their importance for inclusion in the COS (Fig. 2).

After a participant initially rated the importance of an outcome, the survey page was refreshed, and the feedback results of how each stakeholder group rated the outcome were displayed for participants in a bar-chart layout. See the example in Fig. 2.

With the graphs displayed, the participant could change the rating they gave that outcome based on how other stakeholder groups rated the outcome, or they could keep their rating the same before moving on to rate the next outcome. Participants could also provide feedback and suggest additional outcomes not already included in the Delphi survey. Text boxes were also included on the last page of the survey for the first ten weeks of both surveys (RTD and MRD). COHESION Steering Group members reviewed additional outcomes from the RTD and the MRD and compared them against already included outcomes.

The degree of consensus changed throughout the RTD survey so participants were invited by email (Fig. 1), to revisit the system to see how the outcome ratings for each stakeholder group had changed (or not) over time.

Participants could save their ratings for an outcome and revisit the RTD survey at a later time. Those that completed a rating for some, but not all outcomes, were emailed and invited to complete the survey and rate the remaining outcomes.

Based on our consensus criteria [9, 13] (Table 1), outcomes below the threshold for inclusion in both survey arms were removed from the RTD survey in week ten of the survey being live. At week ten, 85% of participants had completed the Multi-Round Delphi and 83% had completed the RTD.

**Table 1:** Consensus criteria for outcomes in the Delphi surveys

**Consensus Classification**	**Description**	**Definition**
Consensus in (parent-weighted vote)	Consensus that the outcome should be included in the core outcome set	70% or more participants *overall* scoring as 7 to 9 AND < 15% participants scoring a 1 to 3 OR > 70% or more of *parent* group scoring as 7 to 9
Consensus out	Consensus that the outcome should not be included in the COS	50% or fewer participants scoring as 7 to 9 in each stakeholder group
Neither consensus in nor consensus out (consensus undetermined)	Uncertainty about the importance of the outcome, so retain for next round	Anything else

#### Multi-Round Delphi method

The MRD survey comprised three rounds. Participants were asked to rate the outcomes on the same 9-point Likert scale as the RTD survey participants. The first round presented the same 87 outcomes to participants as those in the RTD. The five additional outcomes were added to round 2 of the survey based on stakeholder feedback. In round 1 of the Multi-Round survey, participants were asked to rate the outcomes and could suggest additional outcomes that were not already included in the Delphi survey.

In round 2 of the survey, participants were shown the stakeholder group ratings for each outcome in bar chart format. As in Fig. 2, the proportion of participants in each stakeholder group, rating each outcome on a 9-point Likert scale, were presented to participants for each outcome. Participants in round 2 could change their rating of an outcome or keep it the same based on the knowledge of other stakeholders’ responses. Participants were also asked to rate the new suggested outcomes for the first time in this round.

Participants who fully completed round 1 were invited to complete round 2. Similarly, those that completed round 2 were invited to complete round 3. In rounds 2 and 3 of the Multi-Round survey, participants were shown how the stakeholder groups rated the outcomes in the previous round. The consensus criteria (Table 1) were used to evaluate the consensus rating for the outcomes in all rounds.

At the end of both surveys (Multi-Round and RTD), outcomes meeting the consensus criteria ‘consensus in’ and ‘neither consensus in nor consensus out’ were carried forward to online consensus meetings for further discussion and voting with representative groups of stakeholders (parents/caregivers, healthcare providers and researchers/academics) to decide on the final COS [9]. Outcomes meeting the ‘consensus out’ criteria were also verified as being excluded by participants at the consensus meetings.

### Recruitment

As stated in our protocol, we sought to recruit at least 180 participants [13]. Stakeholders were recruited through parent support networks, charities, email invitations to experts who have published on neonatal encephalopathy, interest groups (Newborn Brain Society, and other societies/organisations listed in our protocol [9], those who had participated in previous similar research, and social media (Twitter and Facebook).

For Twitter, a recruitment video was developed (see: https://www.youtube.com/watch?v=lRW7n8HtczQ), and the survey link and information were posted. Other participants were recruited through email invitations, including the survey link and survey information. Those recruited through emails were also asked to forward the email invitation to colleagues who may be interested in taking part in the study.

### Randomisation

When participants clicked on the survey link, they initially entered a survey where the Participant Information Leaflet (PIL) was provided. Participants were asked to select which role identified them best for this project. After consenting to participate in the survey (Additional file 1: Appendix 2), participants were immediately randomised to either the MRD or the RTD in a 1:1 ratio, using computer-generated blocked randomisation with random block sizes of 4, 6 and 8, and stratified by stakeholder group (parents/caregiver, healthcare providers, or researchers/academics).

### Statistical analysis

#### Outcomes

##### Primary outcome

We compared the list of prioritised outcomes at the close of both the RTD and MRD surveys [9].

##### Secondary outcomes

1. Feedback effect

We assessed the ‘feedback effect’ (as defined by Gnatzy et al.) to determine if and to what extent the stakeholder response feedback that was provided to participants changed how they rated the importance of an outcome. In the RTD, participants could see feedback in real-time. In contrast, in the MRD, participants were only given feedback on how stakeholders rated each outcome in the previous round at the beginning of rounds 2 and 3.

To assess the ‘feedback effect’, we used a mixed-effects model, with the difference between participants’ final and initial scores for each outcome as the outcome variable, and deviation in participants’ final score from the final mean score, the type of Delphi survey (coded where 0 = MRD and 1 = RTD), and an interaction effect between these two terms as the predictor variable. As participants had more than one rating in multiple outcomes, we also included participant identifier as a random effect. We did not include an ‘outcome’ term in the model, thus assuming that mechanisms influencing outcome ratings are exchangeable and equal between all outcomes.

This model, therefore, examines the relationship between the ‘correction’ in participants’ scores relative to their ‘deviation’ from the group average final score. A significant interaction effect would indicate a difference in the feedback effect between the two Delphi approaches.

2. Iteration effect

In the MRD, the participants could only re-rate outcomes in round 2 and round 3. In the RTD, participants could re-rate outcomes as often as they wished during the period of the live survey. To describe this, we calculated the number of participants in the first and final (third) round of the MRD, and the number of participants providing valid scores at both the first and final round, to establish the attrition rate in the MRD. For the RTD, we tabulated the number of times participants re-rated scores to investigate how many, and how many times, participants re-rated outcomes.

To determine if there was a difference in the convergence process (i.e. agreement of ratings among stakeholders based on the number of rounds or revisits) between the RTD and the MRD surveys, we conducted three standard deviation tests (using Stata’s -sdtest- command) to assess changes in variance in the approaches. Firstly, we tested if the standard deviation of the first and final scores differed in the multi-round dataset alone. We then repeated this test in the RTD dataset. Finally, we then tested if the standard deviation of the difference between the first and final scores (in those that re-rated their score at least once) differed between the MRD and RTD datasets, a significant difference therefore indicating that the convergence process between the two approaches was distinct.

3. Initial condition effect

The initial condition effect examines if there were differences in how participants recruited early to the trial amended their ratings of the outcomes, compared to those recruited later. This analysis is only relevant to the RTD, as the MRD requires all participants to examine and re-rate the scores, therefore we only used the RTD dataset for this analysis.

We ran two models: Firstly, as the first 17-recruited participants populated the survey with scores without being provided feedback on the groups’ scores until after the 18^th^ participant had submitted their initial scores, we created a dichotomous variable to explore if the ‘correction behaviour’ [6] (i.e. the difference between initial and final rating) differed in these 17 compared to the rest of the sample, and relative to their initial score’s difference from the group final mean score (i.e. their ‘deviation’).

To test this, we used a mixed-effects model, with difference between initial and final rating (i.e. ‘correction’) as the outcome; the difference between initial and group mean final score (i.e. ‘deviation’), recruitment order (coded 0 where the participant was one of the first 17 recruits, and 1 for those thereafter), and an interaction effect between these two terms as predictor variables. Again, we included participant identifiers as a random effect to account for the repeated observations among participants.

Given that coercing recruitment order into a dichotomous variable as we did is a relatively strong assumption that may also have reduced power in the analysis, we ran a sensitivity analysis that repeated the initial condition model. We used recruitment order as a continuous variable (running from 1 to 111 from first to final participant). This model again was a mixed-effects model, with ‘correction’ as the outcome, and ‘deviation’, recruitment order, and a deviation-by-recruitment order interaction effect as predictor variables, and participant identifier as a random effect.

Participants that did not alter their score between initial and final ratings were excluded from this analysis as the majority of these participants did not revisit their score rating and therefore are irrelevant to the initial condition model.

All analyses were undertaken using Stata, version 17.0 [16]. Statistical significance was assessed assuming a type-I error rate of 5%, and 95% confidence intervals were produced for model coefficients.

## Results

### Deviations from the protocol

In our protocol, we had stated that participants who rated an outcome six or more points differently (on the 9-point Likert scale) from those in their stakeholder group would be given the opportunity to explain their rating. However, based on changing consensus over time, we did not follow up with individual participants on their individual ratings.

Due to the risk of recall bias [17–19] among the RTD participants by the time the MRD had closed, we took the decision not to send the USE (Usefulness, Satisfaction and Ease of use) questionnaire to participants [20, 21] as planned in our protocol.

### Sample

**Fig. 3:**
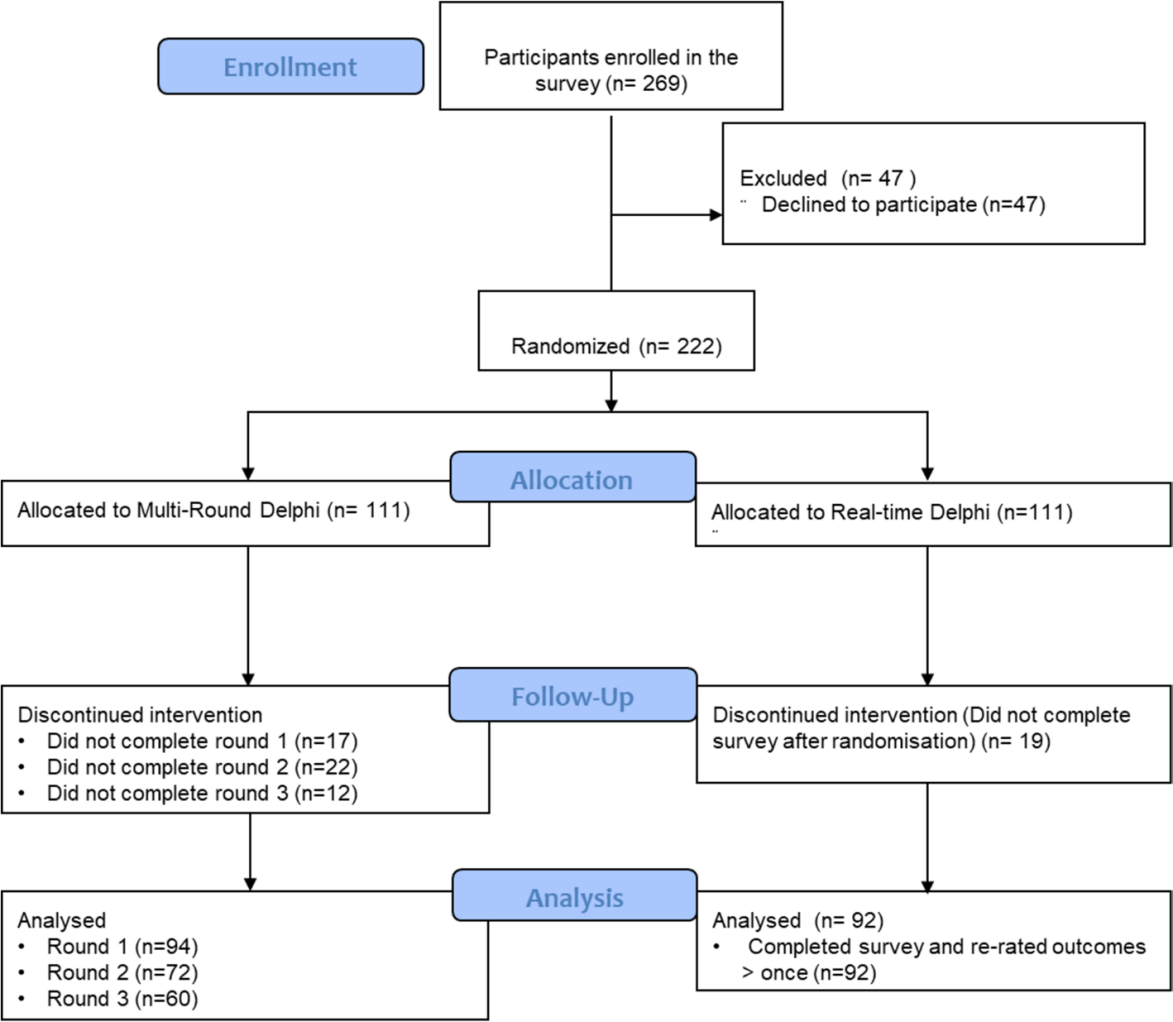
CONSORT Flow Diagram of the progress through the phases of a parallel randomised trial of two groups

In total, 269 participants accessed the survey link. Of these, 222 completed the initial survey and were randomised to either the MRD (*n* = 111, 50 parents/caregivers, 50 healthcare providers, 11 researchers/academics), or the RTD (*n* = 111, 49 parents/caregivers, 51 healthcare providers, 11 researchers/academics) participants. In the RTD, *n* = 92 (83%) participants (38 parents/caregivers, 44 healthcare providers, 10 researchers/academics) fulfilled the Delphi criteria (i.e. re-rating the outcomes at least once after seeing stakeholder rating feedback). In the MRD, 94 participants (85%) completed round 1 (41 parents/caregivers, 43 healthcare providers, and 10 researchers/academics), 72 participants (65%) completed round 2 (31 parents/caregivers, 33 healthcare providers, 8 researchers/academics), and 60 participants (54%) completed round 3 (27 parents/caregivers, 26 healthcare providers, and 7 researcher/academics) (Fig. 3).

### Findings

#### Primary outcome

There were differences in how the outcomes were prioritised between the two survey types at the close of both surveys.

At the end of the 3^rd^ round of the MRD, 50 outcomes met the criteria for ‘consensus in’, 12 for consensus out and consensus was undetermined for 12 outcomes. At the end of the RTD survey, 65 outcomes met the criteria for ‘consensus in’, six for ‘consensus out’, and consensus was undetermined for 21 outcomes.

At the end of both the RTD and MRD surveys, of the 92 outcomes (87 outcomes at the beginning of the Delphi surveys and the five additional outcomes suggested by participants), 48 outcomes were rated as ‘consensus in’ by both surveys and a further six were rated as ‘consensus out’ in both surveys. Of the remaining 38 outcomes, 13 outcomes were rated as ‘neither consensus in nor consensus out’ by both surveys; 19 outcomes were rated as ‘consensus in’ by one survey but ‘neither consensus in nor consensus out’ by the other survey; six outcomes were rated ‘consensus out’ by one survey but ‘neither consensus in nor consensus out’ by the other survey (Table 2).

**Table 2:** Results of outcome ratings from each survey type based on consensus criteria; ‘in’ relates to outcomes rated as ‘consensus in’; ‘out’ relates to outcomes rated as ‘consensus out’; ‘undetermined’ relates to outcomes rated as ‘neither consensus in nor consensus out’

Outcome	Multi-Round	Real-Time
Brain injury on imaging	In	In
EEG abnormalities	In	In
Intracranial haemorrhage	In	In
Severity of encephalopathy	In	In
Absence of neonatal reflexes	In	In
Gag reflex (absence)	In	In
Swallow (absent)	In	In
Neonatal seizures	In	In
Normal tone	In	In
Need for neonatal resuscitation	In	In
Oxygen requirement	In	In
Need for mechanical ventilation	In	In
Respiratory distress	In	In
Ability to breathe normally and unaided	In	In
Need for tube feeding	In	In
Oral feeding ability	In	In
General gross motor ability	In	In
General fine motor ability	In	In
Need for physiotherapy	In	In
General cognitive ability	In	In
Normal memory	In	In
Child mental health	In	In
Visual impairment	In	In
Speech delay	In	In
Ability to make noises/verbalise	In	In
General communication ability	In	In
Psychological development	In	In
Self-esteem	In	In
Behavioural issues	In	In
Cerebral palsy	In	In
Epilepsy	In	In
Survival	In	In
Suffering	In	In
Full recovery from acute illness	In	In
Quality of life of the patient	In	In
Readmission in childhood	In	In
Adverse events	In	In
Hypoxia	In	In
Multi-organ dysfunction	In	In
Emotional impact on parents	In	In
Parental involvement in care	In	In
Parental attachment with their baby	In	In
Parental sense of loss of normal	In	In
Uncertainty for child’s future wellbeing	In	In
Parental psychological impact of NICU experience	In	In
Impact of child’s condition on parent’s relationship	In	In
Financial burden of healthcare costs of care for an infant on parents	In	In
Effective communication	In	In
Parental ability to work	In	Undetermined
Feeding intolerance	In	Undetermined
Biomarker evidence of brain injury	Undetermined	In
Myocardial dysfunction	Undetermined	In
Cardiac ischaemia	Undetermined	In
Need for extracorporeal membrane oxygenation	Undetermined	In
Persistent pulmonary hypertension	Undetermined	In
Pulmonary haemorrhage	Undetermined	In
ADD	Undetermined	In
Heightened sensory sensitivity	Undetermined	In
Hearing impairment	Undetermined	In
Requirement for antiepileptic drugs at discharge	Undetermined	In
Need for occupational therapy	Undetermined	In
Growth	Undetermined	In
Requirement for analgesics	Undetermined	In
Sepsis	Undetermined	In
Duration of neonatal stay	Undetermined	In
Need for multiple operations	Undetermined	In
Impact on family decision to have other children	Undetermined	In
Abnormal changes in heart rate or rhythm	Undetermined	Undetermined
Coagulopathy	Undetermined	Undetermined
Thrombosis	Undetermined	Undetermined
Hypotension	Undetermined	Undetermined
Need for inhaled nitric oxide	Undetermined	Undetermined
Necrotising enterocolitis	Undetermined	Undetermined
ADHD	Undetermined	Undetermined
Pyrexia	Undetermined	Undetermined
Poor renal function	Undetermined	Undetermined
Hepatic dysfunction	Undetermined	Undetermined
Metabolic acidosis	Undetermined	Undetermined
Pneumonia	Undetermined	Undetermined
Hypoglycaemia	Undetermined	Undetermined
Sleep disorders	Out	Undetermined
Hypertension	Out	Undetermined
Haematological variables	Out	Undetermined
Healthcare costs	Out	Undetermined
Electrolyte disturbance	Out	Undetermined
Impact of child’s condition on Neonatal Intensive Care Unit experience on wider family (stress, disappointment, sadness, grief, etc.)	Out	Undetermined
Lung air leaks	Out	Out
Need for surgical treatment of gastroesophageal reflux disease	Out	Out
Meconium passage	Out	Out
Ability to undertake sport	Out	Out
Jaundice	Out	Out
Hyperglycaemia	Out	Out

One outcome (ability to undertake sport) was rated ‘consensus out’ in the first round of the Multi-Round survey and after recruitment had stopped for the Real-Time survey. This outcome was removed in round 2 of the MRD and after recruitment had stopped in the RTD. The other five outcomes rated as ‘consensus out’ (lung air leaks, need for surgical treatment of gastroesophageal reflux disease, meconium passage, jaundice, and hyperglycemia) were rated as ‘consensus out’ by the close of both surveys.

#### Secondary outcomes

##### Feedback effect

**Fig. 4:**
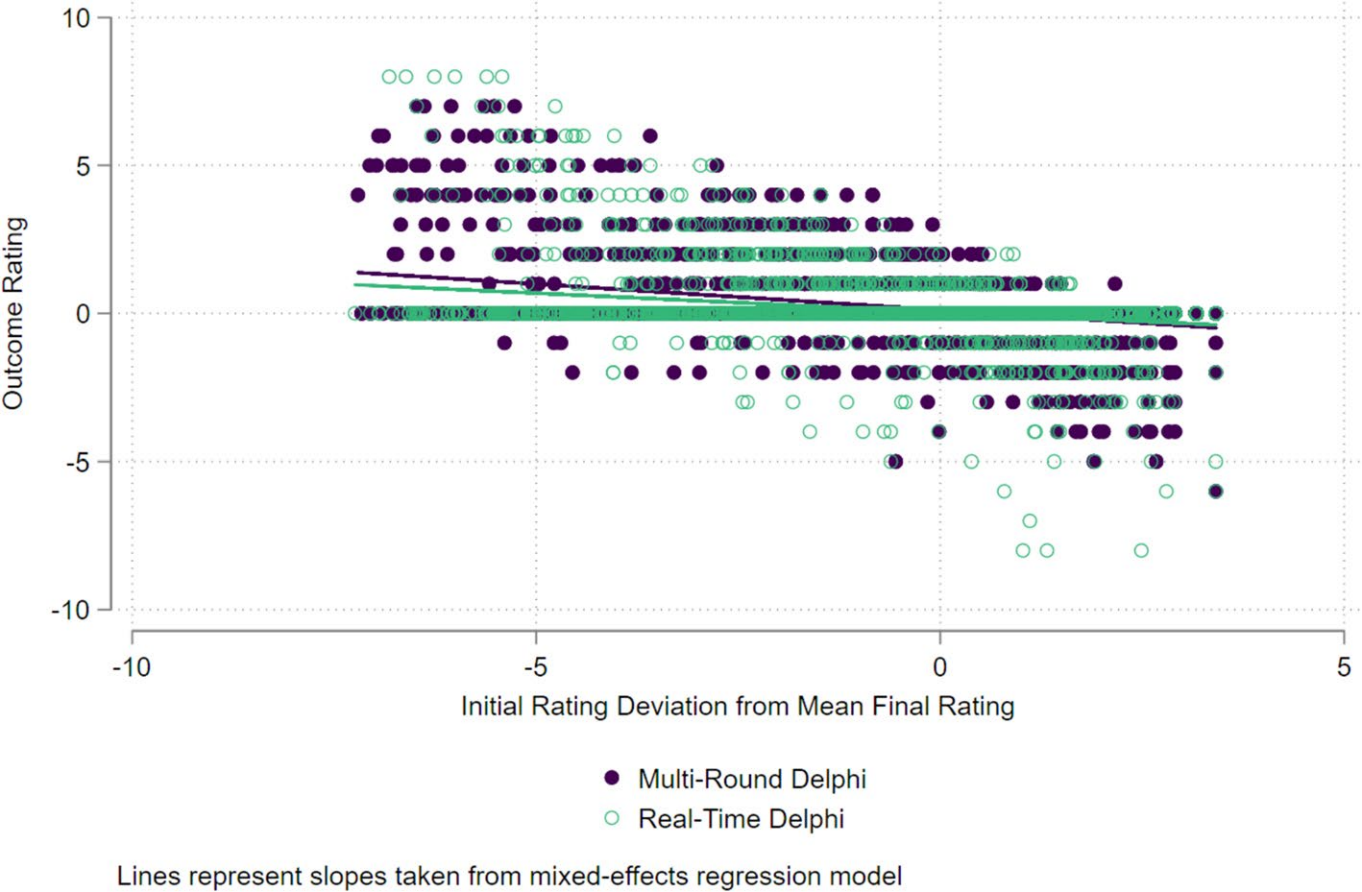
‘Feedback effect’ model indicating slopes of the difference between initial and final rates of participants in the two survey methods

For the feedback effect, our model found a difference in ‘feedback effect’ between the two survey types. As shown in Fig. 4, while the difference between the slopes (the interaction effect in the model) was statistically significant (*b* = 0.05; 95% CI 0.03 to 0.06; *p* < 0.001), the magnitude of the slope is trivial — it suggests that participants in the RTD corrected themselves towards the group final score on average by 0.05 Likert scale points less than in the MTD, an arguably minor amount.

##### Iteration effect

In the MRD survey, 60 participants completed all three rounds and re-rated or kept the same rating for an outcome. In the RTD survey, all participants revisited the survey at least once, with the maximum number of revisits being 29 times.

**Fig. 5:**
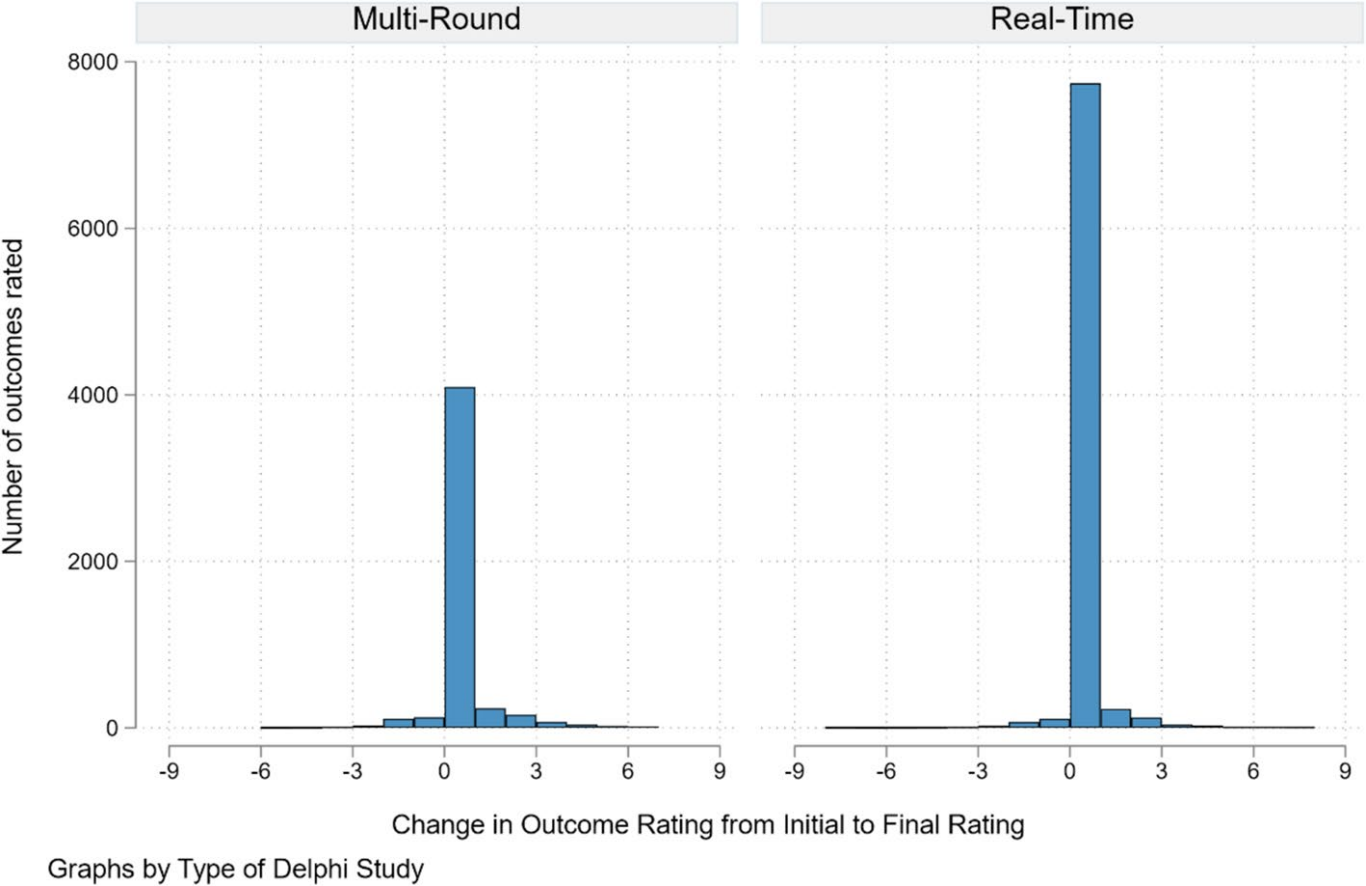
Histograms showing the distribution of the difference between initial and final scores in all participants in both survey types

**Fig. 6:**
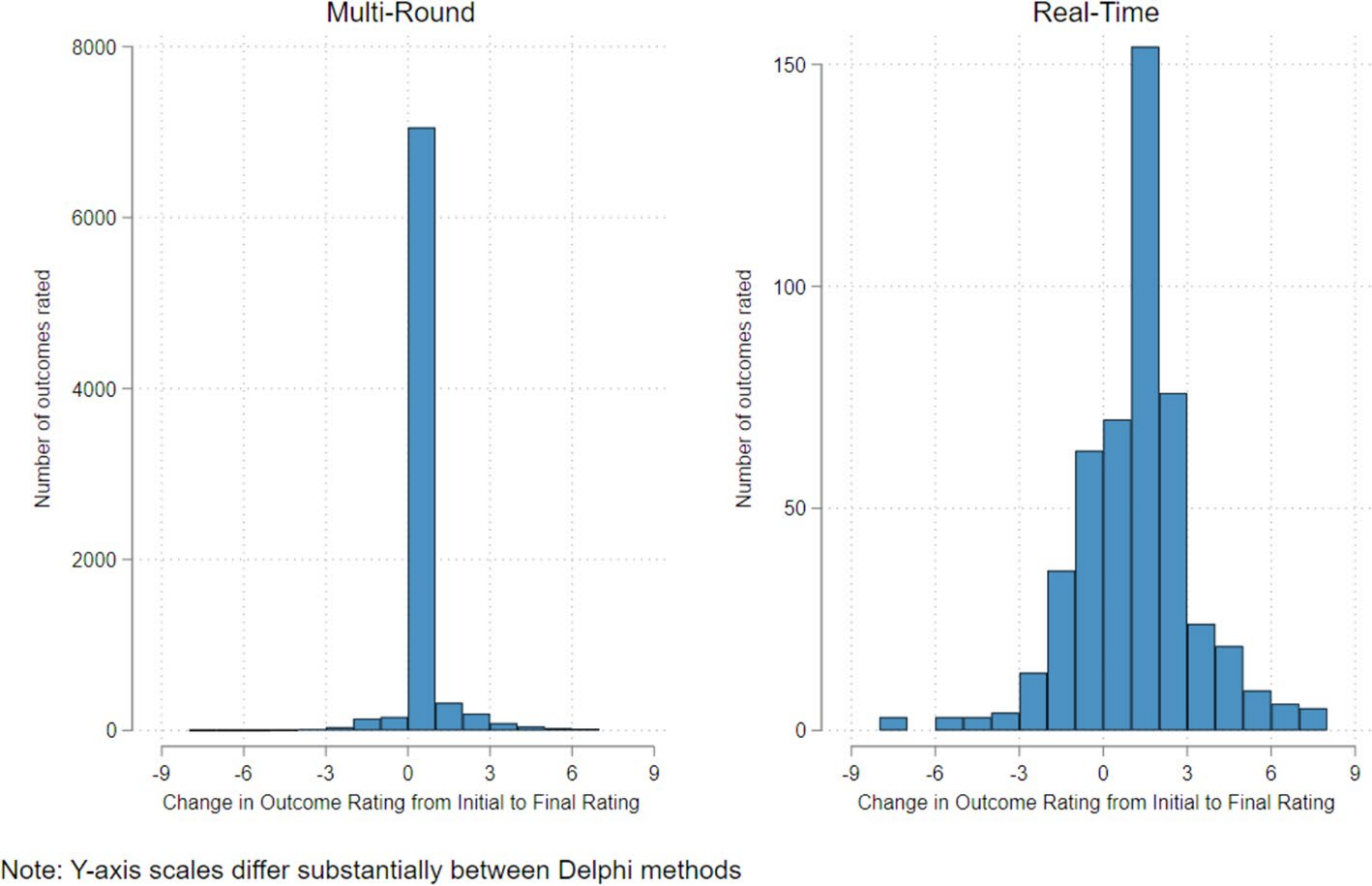
Histograms showing the distribution of the difference between initial and final scores in those participants that revisited their initial rating at least once

For the iteration effect, we found that the standard deviation of the distribution of rating scores in the MRD dataset went from 2.17 initially to 2.08 at the final rating (*p* =  < 0.001), indicating that variation in ratings significantly reduced over time. The standard deviation over the entire RTD dataset (i.e. everyone who completed the RTD survey) reduced from 1.92 initially to 1.87 at the final rating (*p* = 0.06), indicating that the variance reduced, but not significantly, overall. However, for those who changed their rating based on stakeholder response feedback, the standard deviation went from 2.07 initially to 1.68 at the final rating (*p* =  < 0.001), indicating that the variance reduced significantly. When looking at whether the variances differed between the MRD and the RTD, the standard deviation of the difference between the initial and final ratings for the MRD dataset is 0.82 and is 0.72 for the RTD dataset (*p* < 0.001), indicating that there was reduced variation in score changes in the RT dataset (Fig. 5). However, when restricted to only those who changed their ratings (Fig. 6), the standard deviation of the difference between initial and final ratings for the MRD dataset is 0.82. For the RTD dataset, the standard deviation of the difference is 2.15 (*p* < 0.001), indicating that, in the RTD dataset, while participants generally did not change their scores much between ratings, when they did decide to alter their score, they did by a more substantial degree than in the MRD.

##### Initial condition effect

**Fig. 7:**
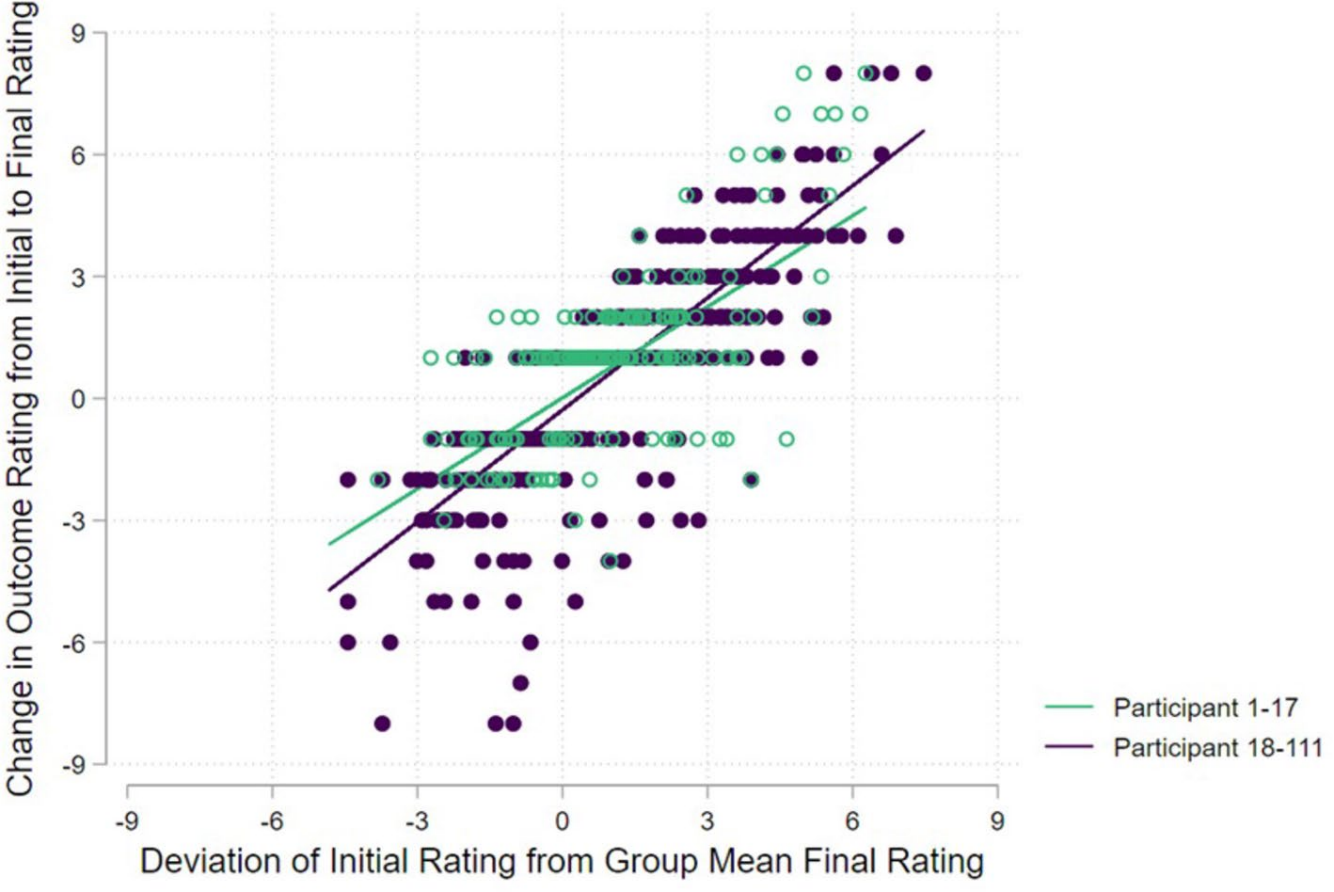
Comparison of the difference (deviation) between initial score of participants and the average final score, and the difference between initial and final scores, for those participants that who altered their score between initial and final rating only. Plotted lines are slopes taken from mixed effects regression model

Of the initial 17 participants’ scores, 93.3% remained unchanged between the initial and final rating. For the 18^th^-recruited and later participants, 85.9% of scores remain unchanged. In the model excluding participants with unchanging scores between the initial and final rating, we found a significant interaction effect between the degree of correction relative to deviance from the group mean score, and whether participants were recruited early (in the first 17) or later (the 18^th^–111^th^ participants), with the later-recruited participants having a steeper slope (*b* =  − 0.10; 95% CI − 0.28 to − 0.07; *p* = 0.001), inferring that earlier participants correct themselves less towards the final mean rating (Fig. 7). We found a similar effect in the alternative model, which considered recruitment order as a continuous variable, again with a significant interaction effect (*b* =  − 0.0021; 95% CI 0.006 to 0.0036; *p* = 0.01), and again inferring that earlier participants correct themselves towards the final mean to a lesser extent than the later-recruited participants. Given the coefficients in this latter model were expressed per participant, we extracted the predicted coefficients at the minimal, maximal, lower and upper quartile, and median recruited participants (i.e. 1^st^, 28^th^, 56^th^, 84, and 111^th^ participants) (Table 3). The predicted coefficients approach 1.0 as recruitment moves towards the final participant, indicating that by the final (111^th^) participant, the degree of correction towards the group mean final score is almost exactly the same as the degree of deviation from that score.

**Table 3:** Predicted coefficients (inferring correlation between correction towards the final mean score and initial score deviation from the final score) taken from the initial condition model, at different points in the trial recruitment order

**Recruitment order**		***b* coefficient**	**Lower 95% CI**	**Upper 95% CI**
Min	1^st^ participant	0.78	0.69	0.86
25^th^ centile	28^th^ participant	0.83	0.78	0.89
median	56^th^ participant	0.89	0.85	0.94
75^th^ centile	84^th^ participant	0.95	0.88	1.02
Max	111^th^ participant	1.01	0.91	1.11

## Discussion

### Primary outcome

Across both the MRD and RTD, 67 (73%) of the outcomes were rated the same: 48 (52%) outcomes were rated as ‘consensus in’ by both surveys; 6 (7%) outcomes were rated as ‘consensus out’ in both surveys, and 13 (14%) outcomes were rated as ‘neither consensus in nor consensus out’ by both surveys. Thus, 67 (73%) of the outcomes were rated the same across both survey arms. There were differences in the way 25 (27%) outcomes were prioritised between the two survey types. The disparities in 19 (76%) of these 25 outcomes were whether outcomes were rated important (4–6 on the Likert scale), or critical (7–9 on the Likert scale) for inclusion in the COS. For the other six outcomes, the disparities were whether the outcomes were rated as not important (1–3 on the Likert scale) or important (4–6 on the Likert scale). All outcomes that were voted ‘consensus in’ or ‘neither consensus in nor consensus out’ by both or either survey, were carried forward for discussion at our consensus meetings.

### Secondary outcomes

#### Feedback effect

The results of the ‘feedback effect’ analysis indicate that the RTD survey results in marginally less correction i.e. changing of rating scores by participants (0.05 fewer rating points, out of 9) following feedback than the Multi-Round survey. This is statistically significant but arguably meaningless, given the difference is a fraction of a point. We believe that the presence of a ‘feedback effect’ shown in our analysis, compared to no effect in the Gnatzy et al. [6] results may be due to our larger sample size rather than a large effect being present.

#### Iteration effect

In the Multi-Round dataset, the variance in ratings reduced between rounds indicating an ‘iteration effect’ (i.e., the converging of scores). In the RTD dataset, most outcome ratings did not change over time, so the variance overall did not reduce, indicating no ‘iteration effect’ overall. However, in those outcomes that were re-rated in the RTD dataset, the variance was reduced substantially. This suggests that most experts in the RTD were happy with their initial ratings, and where they were not, they amended them substantially. There did not seem to be much advantage in participants having to re-rate their outcomes over subsequent rounds, as convergence in the RTD dataset, where re-ratings were allowed but not enforced, may be better.

At first glance, the ‘feedback’ and ‘iteration effect’ results seem slightly contradictory. The slope of the ‘feedback effect’ (Fig. 4) model suggests the RTD participants changed their initial rating to their final rating less than participants in the MRD. The ‘iteration effect’ appears to indicate the opposite. However, most participants in the RTD did *not* amend their ratings over time, but those that did amend their ratings changed their ratings more than participants in the MRD.

#### Initial condition effect

Where participants did amend their outcome rating, they amended the rating towards the group mean, but the degree to which they corrected their scores depended on their recruitment order. Earlier participants corrected themselves less to the final group mean score than later participants. This may partly be due to the fact that the first 17 participants did not have access to the stakeholder response feedback upon first completing the survey. However, whilst we did see what appears to be an ‘iteration effect’ in those participants that changed their score, the overwhelming majority of participants in the RTD survey did not amend their rating of an outcome after the initial rating, inferring that the ‘initial condition’ effect is not applicable the majority of the time.

The RTD approach allowed participants to engage with the survey without waiting for the survey administrator to evaluate the stakeholder responses and generate the feedback graphs. It also freed up time for the survey administrator to monitor respondent rates and stay up-to-date with participant reminder emails.

### Limitations

A source of bias (performance bias and detection bias) in this randomised trial is the lack of blinding of survey participants and lead researcher. However, it was necessary to provide instructions to participants on how to complete the survey and for the lead researcher to access the survey database to extract data for analyses, monitor participation and provide updated reports and other tasks. A further limitation was that the sample size was guided by the need to include stakeholders to develop the overall COS rather than the trial itself.

Data are missing from the analysis of the feedback effect, iteration effect and initial condition effect. These data relate to the five outcomes participants suggested in the survey. These outcomes were not rated and/or re-rated by all participants, so the rating trends for these outcomes were omitted from the analysis.

Our analysis did not consider contextual differences between outcomes, assuming that the underlying mechanism influencing all outcomes was homogenous and, therefore could be omitted from the analysis (i.e. we did not include an ‘outcome’ term or interaction effects by the outcome in the analyses). Whilst this is a relatively strong assumption, it follows the analysis approach used by Gnatzy et al. [6] and also simplifies the analysis and interpretation of the feedback, initial condition, and iteration effects (given that we were considering over 90 outcomes simultaneously).

In terms of the COS itself, we do not know what the implications in the final COS would be if we had just run one survey approach. However, by conserving all outcomes rated as ‘undetermined’, we ensured that important outcomes were not removed and that they could be discussed further at the consensus meetings. We began with a large menu of potential outcomes, and neither Delphi method alone weaned the list down to a manageable number of outcomes to propose in a COS.

## Conclusions

### Importance and relevance of this study

There is a relatively low uptake of COSs by trialists [22] despite the need to reduce heterogeneity in outcomes reported in trials for various conditions. Researchers are more likely to use the COS when contributing to its development [1]. Therefore, it is important to ensure optimal participation in each stage of the COS development, including the Delphi survey stage. The length of time to complete multiple rounds of a Delphi [5] has been recognised as an impeding factor to participation; therefore, an approach that takes less time may help to improve stakeholder participation in the development of COSs.

Our findings show that compared to a MRD, a commonly-used approach in COS development [1], there did not seem to be an advantage to making participation in multiple rounds of a Delphi survey obligatory. Overall, convergence in the RTD was greater, where revisits were allowed but not compulsory. Although we tried to avoid an initial condition effect by populating the RTD survey with data from 17 participants before the survey went live to the public, an initial condition effect was seen in the small proportion of participants that did amend their score after the initial rating.

Advantages of using the RTD method include reduced time to complete the survey overall, as the RTD was live for 14 weeks compared to the MRD being live for 20 weeks.

Additionally, the RTD achieved better convergence on outcome ratings. As many COSs were developed five or more years ago (see COMET database: https://www.comet-initiative.org/), there should be a plan to revisit these COSs to ensure the outcomes are still relevant given the rapid evolution of evidence in many conditions. A RTD method may confer benefits in reviewing existing COSs as a quicker alternative to repeating multi-rounds of a traditional style Delphi.

## Supplementary Information

**Additional file 1: Appendix 1.** CONSORT 2010 checklist of information to include when reporting a randomised trial (Chapter 6). **Appendix 2.** Consent questions for participants in the Surveylet (Calibrum) software.

## Data Availability

All datasets used and/or analysed during the COHESION study will be held by COHESION team at f.quirke1@nuigalway.ie.

## Abbreviations

RTD: Real-Time Delphi
MRD: Multi-Round Delphi
